# Service innovation in small neighborhood family firms: An advanced approach to enhance employee's performance through social and psychological rewards

**DOI:** 10.3389/fpubh.2022.984848

**Published:** 2022-08-12

**Authors:** Muhammad Waqas Sadiq, Javaria Hameed, Chunhui Huo, Muhammad Ibrahim Abdullah

**Affiliations:** ^1^Business School, Liaoning University, Shenyang, China; ^2^Department of Management Sciences, Commission on Science and Technology for Sustainable Development in the South (COMSATS) University Islamabad, Sahiwal, Pakistan; ^3^Department of Management Sciences, Commission on Science and Technology for Sustainable Development in the South (COMSATS) University Islamabad, Lahore, Pakistan

**Keywords:** social rewards, psychological rewards, small family firms, employee satisfaction, service innovation

## Abstract

This research study focuses on the employee's job performance of private small firms during the post COVID-19 situation. After the COVID these small family firms try to regain their business, but their efforts are not that much successful. This situation creates a financial crisis in these firms, and they are unable to provide sufficient monetary rewards to their employees. This situation creates unrest among the employees of these small firms. To manage this issue, social rewards and psychological rewards played their role. The study uses a causal research design with a correlational study design in a non-contrived environment. Minimal researcher interference has been assured. AMOS 24 has dealt with the mediation in study design with bootstrap methodology. The study was conducted on 250 employees of different private small family firms across Punjab province using a proportionate stratified sampling technique. A study's finding suggests that top management enhances employee performance in their organizations by introducing the organization's psychological rewards. In contrast, introducing social rewards does not significantly impact employee performance while considering satisfaction and motivation as a mediating variable.

## Introduction

Job creation is the top priority of policymakers in every developing country. With many countries experiencing rapid growth in the population, governments must create a significant number of employments to keep up with labor demand, and even more, jobs are required for economic growth. Small firms have created more and large number of employments than larger firms. This helps to grow the economy of the developing countries (1). Small firms need skilled labor and loyal employees for effective production. If the employees or laborers are satisfied with their jobs, their performance would have increased. In this era, it is hard to find skilled and loyal labor. Firms must provide the best working atmosphere for their employees, so they work in a healthy work environment that satisfies them with their jobs. Social recognition, acceptance, and praise significantly impact the employee has good reputation and motivation to have such social approvals (2).

It is essential to understand that retaining essential employees serves what purpose in the company while maintaining their performance up to the maximum level. According to earlier research, the typical business loses $1 million for every ten managers and professionals leave. More than 2 years of compensation and benefits are required to replace an exempt employee who is terminated for cause (3). When a key employee departs from a firm, valuable knowledge is lost that might significantly impact the company's financial health. Through the use of this information, customers' needs and expectations are satisfied. The term “knowledge management” refers to creating, gathering, and using data to benefit a company's operations. While information is recognized as an organization's most valuable asset today, many firms lack the mechanisms necessary to preserve and use its value (4, 5). Companies must actively participate in knowledge management rather than depending on the premise that employees are acquiring and using information and that knowledge sources are freely available. Firms are designing systems that use the value of information to sustain their competitive advantage. It's easy to see the consequences of losing individuals with access to critical information. Human capital and knowledge management are based on the premise that workers are a company's most crucial asset due to their diverse range of skills, information, and perspectives. Skills, knowledge, and experiences are regarded as capital because of their ability to boost output (6, 7). To the extent that more resources are allocated to a machine, it is more probable that it will be productive. This is according to the human capital idea. According to human capital theory, business investments in human capital are more profitable and more likely to last for a longer period of time. Keeping your personnel is critical to getting the most return on your investment. Human capital theory uses an employee's length of service as a proxy for their job-related knowledge or ability. A person's work-related knowledge or ability affects everything from pay and progress to job type (8, 9). People who have worked for an organization for a long period of time have a high level of intellectual capital. It was said that intellectual capital is “competence multiplied by commitment,” which indicates that a company's knowledge, talents, and traits are amplified by the will to work of everyone inside it. Recognizing employees' commitment to a firm and creating an environment that encourages long-term commitment will be crucial in the future years. Individual knowledge that has been developed through time must be preserved or organizations will continue to lose valuable intellectual capital. A company's deep awareness of its sector is one of the most important factors that contribute to today's success in the global marketplace (10, 11).

The small firms act like cushions to economic shocks for the country's economic stability. In Pakistan, primarily small firms remain small a few of them expand. In this study, the main focus is on the employee productivity and satisfaction by giving them social and psychological rewards. The social exchange theory argues that social behavior is motivated by the ratio of social rewards. According to this theory, people involved in social behavior when there is any benefit for them, not only monetary but social acceptance from others (12). COVID-19 has a significant influence on society. The economic conditions are also affected by this situation. In this situation, the firms can only give the salary to their employees. The firms focus on enhancing the employee's productivity by providing them social and psychological rewards that are not monetary. So the firms do not have to invest money in this critical situation.

Many researchers do their research on employee performance in small firms (13). Understand the impact of employee learning on staff motivation (14). Research about the effects on employee performance and satisfaction (15). This study measure job attitude among 80 employees of four different small businesses. Each employee filled a survey form. Employees show clear difference in their job satisfaction.

## Research gap

The gap of this research is that there are two mediator studied in this research. There are millions of other variables that are affecting on employee performance. In previous studies, researchers did not study specific variables like social rewards, psychological rewards, and employee motivation and satisfaction with employee performance, especially in Pakistan, but they studied it with different variables. Other researchers studied employee performance and satisfaction together but they did not study in perspective of social and psychological rewards (16).

## Research objective

The study seeks to achieve the following objectives:

1. Examine the link between employee contentment and productivity.
2. Employee happiness and motivation to understand the practical effects of social incentives on workplace performance.
3. To determine the practical effects of psychological incentives on the performance of employees *via* the pleasure and motivation of employees.
4. Find out what reward is most suited for a small family-owned business.

## Research questions

This research question is more clarified with sub-questions:

1. Is there any effect of social rewards on employee performance in firms?
2. Is there any effect of psychological rewards on employee performance in firms?
3. Is there a correlation between employee happiness and productivity?
4. This research is conducted to answer the following question; what is the impact of social and psychological rewards on employee performance affecting employee satisfaction?

## Significance of the study

To maintain its finest personnel for the long term, every sector uses a combination of financial and non-financial incentives. In this research, small businesses and industries learn how to boost employee happiness and productivity by creating a healthy work environment. This study also helps firms to know that the social or psychological rewards help to attract the employees.

The purpose of this research is to get insight and understanding of employee's minds for their satisfaction by social and psychological rewards to increase their performance. The study's objective is to provide information to small firms to keep employees satisfied and increase their performance by non-monetary rewards.

## Literature review

### Theoretical model development

**Figure 1:**
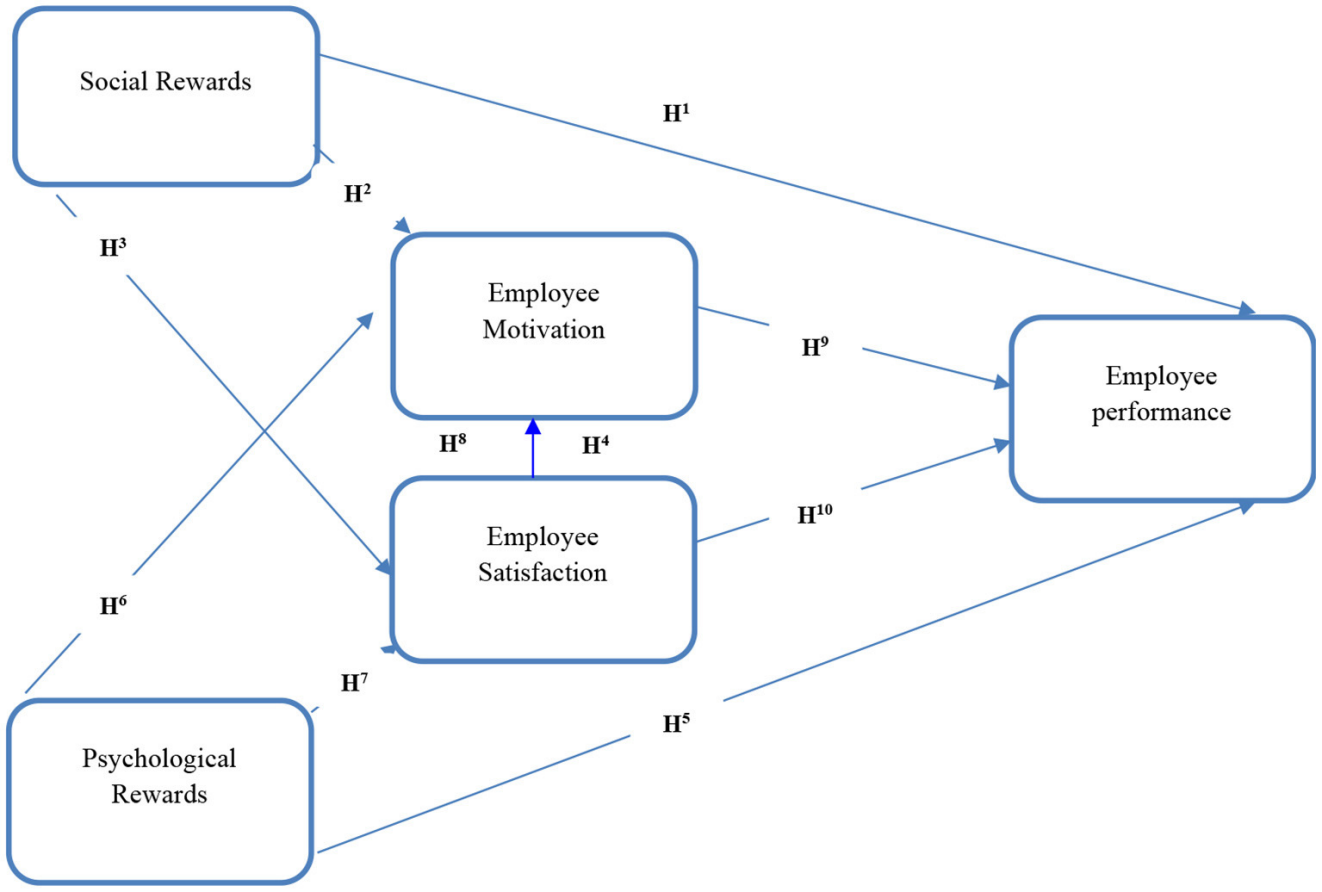
Theoretical model.

Before moving on to the literature review, it is important to understand why these variables need to be studied together and what theories supported this theoretical framework which is depicted in Figure 1, and why could it be considered an important management problem? The theoretical framework used in this study is backed by the Componential Theory of Creativity (17). According to this theory it specifically focuses on the social and psychological rewards given by the organizations, which could ultimately enhance the level of creativity of its employees. This level of creativity could lead toward the improved performance of employees in the organization. This kind of theoretical framework and research work is also supported by different studies (18–20) which focuses upon the need of finding effective non-monetary rewards which could lead toward the enhanced employee performance through motivation and satisfaction.

### Social rewards

It's not always as easy to achieve social objectives as achieving more fundamental physiological ones, like getting enough food. Many of society's aspirations are nebulous and ill-defined. When a person's hunger causes them to seek out cake, they can readily determine whether they achieved their aim or not. As a result, it is less evident how to go about finding a social connection, and whether one has really succeeded is a matter of debate. No crumbs or chocolate-smeared surfaces are left in the aftermath of social aims, in contrast to cake. How might readers connect proximal reward signals to their ultimate goals (21, 22). Previous studies have identified two main problems in understanding how proximate incentives assist individuals attain their ultimate social objectives. The first step is to identify the fundamental social values and incentives that drive ordinary social conduct. What are the underlying principles of human behavior (23, 24). Second, how people's social activities help them achieve their ultimate social objectives, what determines the degree to which an action may attain those goals. Existing research suggests two complementary methods for dealing with each problem. Decontextualizing social incentives to understand how proximal components (i.e., social rewards) influence social actions is the first step they advocate (25). The strength of each fundamental element may be gauged by measuring its value. These elements that remain valued even when separated from the rewarding outcomes they are meant to anticipate serve as the most strong or fundamental drivers of social conduct. Those who cherish the presence of others may seek out ways to see others (e.g., look at photos of their faces) even if they cannot communicate with them directly. Fundamental motivations should be quantifiable even if the final aim is not achieved. These fundamental societal ideals may be identified by decontextualization (26). As a follow-up, they advocate recontextualizing incentives by returning motivation and context to social conduct in order to comprehend how social actions support their ultimate aims. Several variables might affect whether or not a person's purpose is met, such as the social context and the individual's motives. Approaching a smiling friend should strengthen social connection, yet approaching an adversary who smiles could put one in danger. As well as influencing action like approaching a smiling person, context also affects the perceived value of fundamental social elements. For example, a grin from a foe has less value than a smile from a friend. This reassessment illuminates the ultimate societal objectives that a smile's social worth is pointing toward societal behavior and its ultimate social purposes are examined *via* various complementary perspectives. Evaluating people's social values and the forces that may and cannot influence those values is at the heart of both methods. It's possible to get insight into social rewards and the higher-level objectives that motivate them by looking at how our brain's reward system promotes social conduct. Each method is discussed in turn, and the framework's consequences for our knowledge of the building blocks of social incentives, their growth, and their influence on employee performance are discussed (27).

Although social rewards are not tangible, they are generated through social encounters. The social reward dimension refers to many aspects of social conditions and employee work relationships, and it measures the degree of external public recognition and internal social support as felt by employees (28). It also created a pleasant sensation of wellbeing when one feels accepted and belongs during social encounters. Social rewards were typically feelings of wellbeing, enjoyment, and interactions with others (29).

Social incentives are vital in promoting and retaining employee happiness in all industries. It is possible that social incentives might be a key factor in enhancing employee efficiency. If a company is serious about increasing productivity, it has to know how to encourage employees and ensure that they are adequately rewarded for their efforts (30).

Social rewards contribute to a positive sense of wellbeing because one feels like they belong, are recognized by others through social activities, and can feel superior through tasks and social interactions (1). The social reward aspect relates to many aspects of the social environment and employee work relationships. It measures the degree of exterior public recognition and internal social support that workers feel. It also produces a favorable sensation of wellbeing when one feels accepted and belongs in social interactions. The most common social rewards were feelings of wellbeing, enjoyment, and social interactions (6).

**H**^**1**^: Social rewards create a positive impact on employee's performance.

**H**^**2**^: Social rewards mediated by employee motivation positively impact employee's performance.

**H**^**3**^: Social rewards mediated by employee satisfaction positively impact employee's performance.

**H**^**4**^: Social rewards positively impact employee performance through serial mediation of employee motivation and the employee satisfaction.

### Psychological rewards

Employees' wellbeing may be improved in five ways: by providing them with meaningful work, flexibility, a variety of challenges, and a work environment they appreciate. When workers connect with their responsibilities and find them rewarding, their employment is meaningful, according to previous studies. When workers have the freedom to act on their own initiative, it is seen as flexible; on the other hand, it is seen as demanding when employees are given the chance to use their talents (31). Employees will be more engaged in their work if their tasks are fascinating and gratifying. According to early research, a combination of extrinsic and intrinsic incentives has been shown to motivate and maintain effective human capital. Other people or organizations give cash or non-financial incentives to workers, known as extrinsic rewards. An employee's job provides the reward when it comes to intrinsic incentives (32). As a result, psychological benefits result from one's labor when it is relevant and well-executed. Workers' psychological needs are met *via* intrinsic rewards, which are internally mediated and contribute to employees' sense of self. Psychological incentives play a particularly crucial role for workers of small family businesses since they are often paid less than their counterparts in larger enterprises. Small family businesses in developing nations are thought to have the lowest real pay increase budgets and compensation range changes (33). Full-time workers are more likely to be paid less than their private or government colleagues. These findings show the need to research intrinsic incentives in the context of small businesses. There are, however, just a few studies that use quantitative instruments that have been shown to be trustworthy when testing across cultures to determine intrinsic rewards. HR managers who advise small family business workers might benefit from an accurate assessment of intrinsic job incentives (34).

Employees benefit from psychological benefits that strongly link job satisfaction and firm performance. Personality attributes such as confidence, assertiveness, and the ability to see the importance in one's work all contribute to a person's overall wellbeing and fulfillment. For example, an employee's capacity to express a feeling of achievement or duty is a psychological reward, as is the ability to convey a sense of accomplishment to the employee (35).

By giving the psychological rewards to the employees, that are interest of many employees, the satisfaction of the employees toward their job is increased. In this way the employees remain motivated and their performance also enhanced that the firms want. When a task is completed effectively, it is usual for individuals to feel a feeling of accomplishment and satisfaction. In order to keep the good sentiments going, the employee must continue to perform well in his or her job (36). Workplace intrinsic benefits may include things like pride in one's work, respect from superiors and/or coworkers, personal development, increased trust in management, satisfaction from one's job and belonging to a team, and the acquisition of new skills a sense of success. For many people, being free to select the job they wish to do is an innate joy. Incentives have been demonstrated to improve employee productivity in studies (37). In the workplace, psychological reward refers to the benefits that employee receives as a consequence of favorable professional interpersonal interactions with his or her clients, coworkers, or supervisors, such as trust, recognition, and praise. Psychological benefits do not need monetary inputs since they just call for dedication, dedication, and effort on the part of the recipient (38).

Individuals' work attitudes may be positively influenced by psychological rewards, which serve to increase morale. Psychological reward serves as a supply of motivating resources in the pursuit of happiness as a form of innate psychological need. That is, if it is not met, it may have a negative impact on people's subjective wellbeing.

**H**^**5**^: Psychological rewards create a positive impact on employee's performance.

**H**^**6**^: Psychological rewards mediated by employee motivation positively impact employee's performance.

**H**^**7**^: Psychological rewards mediated by employee satisfaction positively impact employee's performance.

**H**^**8**^: Psychological rewards positively impact employee performance through serial mediation of employee motivation and the employee satisfaction.

#### Employee motivation

Motivation's major goal is to make it simpler for people to modify their behavior. Intrinsic motivation is the driving force behind a person's actions toward a certain objective. In a study on employee motivation, the results showed that motivation influenced outcomes such as productivity, performance and persistence. Studies show that motivated employees are more self-driven than their less motivated counterparts because they are more focused on their own independence and autonomy. As a result, highly motivated employees are better equipped to take advantage of the many opportunities for professional advancement that exist. In a similar line, motivated employees are more committed to their jobs than their less motivated counterparts, resulting in better quality work (39).

If you want your dreams to come true, you must have a strong drive to succeed. Whatever influences people's behavior in order to reach a certain objective qualifies as a motivator. Our definition of “motivation” is the means through which an individual's desire to achieve their goals is accounted for, together with their interactions with their environment and the results of that interaction (40). Motivational processes significantly impact a person's overall strength and direction of action. Although motivated behavior happens only in the here and now, its attention is on the future within this time. Workers' work habits are influenced by their degree of motivation at the time. Workplace motivation, whether intrinsic or extrinsic, is critical to employees' wellbeing since it is the fundamental driver behind their attendance. An intrinsic motivation is fueled by a person's interest, pleasure, or enjoyment in a certain activity. Extrinsic motivation occurs when you engage in a task because you like it or find it pleasant (41).

If you want to be inspired, you must know what drives you and what inspires you. Therefore, it is difficult for a person to feel motivated by just partially addressing their needs. One set of demands is fulfilled and another is created; motivation leads to goal-directed behavior; a person behaves in order to achieve their own aims and wants; and there is no one-size-fits-all philosophy or approach to motivation since individuals have varied motivations; Managers need to have an understanding of many different types of motivating elements in order to be effective (42).

Today's competitive business world necessitates that successful organizations find new and innovative methods to motivate and inspire their employees. This is contrary to the findings of earlier research, which reveal that no organization can progress or flourish if its employees are not motivated to do their duties. Every organization's success is closely linked to its employees' motivation, dedication, and perseverance (43). As a consequence, one of the most important jobs or obligations of a leader is to inspire others. An organization's leadership begins with the initial effort to hire a new employee and continues through the whole induction process and every day until the person quits the organization. The importance of leadership to a firm is once again highlighted in the process of bringing in new employees (44). Employee motivation depends on the quality of the boss-employee relationship. Employees who are surrounded by colleagues who exhibit a professional, happy, and polite demeanor are more inclined to follow suit. Organizational culture directly influences employee engagement, productivity, wellbeing, and overall wellbeing in the workplace. Some experts in the field believe that a company's management style affects employee motivation. A lot of individuals disagree on whether or not it's possible to cultivate leaders from scratch. From the discussions on motivation, it's evident that individuals have a broad variety of traits. Many of these effects may be handed down *via* DNA or acquired through exposure to various environmental stressors (45).

The topic of motivation is covered in more depth in the field of organizational behavior, which includes a wide range of models and theories that are pertinent to the topic of motivation. Significant emphasis is placed on the development and advancement of staff members. According to a number of studies, growth is the single most significant incentive for those who attempt to maximize the potential of their staff. It has been discovered that there is an indisputable connection between the motivation of workers, their level of job satisfaction, and their level of commitment to their organizations. The motivation of workers is the single most crucial factor in every company's success, regardless of whether the business in question is public or private. According to the findings of a study that investigated the connection between employee motivation and job satisfaction, businesses that used a variety of motivational programs that centered on three distinct concepts namely, camaraderie, equity, and achievement were judged to be more successful than businesses that either had no “enthusiastic” employees or twice as many of them. Participants in the research numbered 135,000 and came from a wide range of nationalities and cultural backgrounds (46). The researchers that conducted the study to establish the link between employee motivation and employee performance came to the conclusion that an increase in employee motivation leads to an increase in employee performance as a direct consequence of the rise in employee motivation (47).

**H**^**9**^: Employee Motivation creates a positive impact on employee's performance.

#### Employee satisfaction

Employee satisfaction has been examined extensively in various sectors, including army, healthcare, civil service, business, psychology, and sociology, according to a review of the literature. Over time, the concept has evolved. However, no significant implication was produced. It is a complex phenomenon that is influenced by a variety of circumstances.

Employee satisfaction has been shown to have a major impact on employee performance, retention, and turnover. Locke (48) “a pleasant or good emotional state arising from the evaluation of one's employment or job experience” was characterized as employee satisfaction. Negative sentiments reflect dissatisfaction with one's job. The positive sentiments that an employee has about their work are what constitute employee satisfaction.

A company's ability to succeed depends critically on the happiness of its employees. Low staff turnover is associated with high levels of employee satisfaction. Keeping employees happy is thus a top priority for any organization. Amidst economic downturns like the one we're experiencing, employers appear to be ignoring a well-known reality about management: managers know this (49).

The level of employee performance in the company grew as a direct result of the rise in employee happiness. The workers will be pleased and motivated about their employment if they are satisfied with the incentives they are receiving. These benefits will boost their performance if they are satisfied with the awards they are getting (50).

**H**^**10**^: Employee satisfaction creates a positive impact on employee's performance.

#### Employee performance

The term “performance” refers to the result that knowledgeable individuals working in a certain environment are able to produce. The productivity and production of an individual as a direct consequence of their progress is referred to as that person's employee performance. The effectiveness of an organization is directly proportional to the performance of its employees. When conducting a performance review of an employee, the things that the individual does and does not do are considered. An employee's performance is evaluated based on the quality and quantity of their production, their level of attendance at work, how accommodating and helpful they are, and how quickly their output is completed. The findings of an investigation into individual performance, which was carried out by, suggest that it is impossible to verify individual performance (51). In addition to this, he claims that if an employee's performance can be measured, then employers have the ability to provide direct bonuses and incentives depending on the individual's level of success.

Companies will go to tremendous efforts to ensure that their clients are happy, but they will not go to the same measures to ensure that their staff are happy. On the other side, customers won't be happy until and until staff members are pleased both before and after they place their orders. Because if workers are content, they will put in more effort, ultimately leading to contentment on the part of the company's clients.

When employees are inspired, they put more effort into their work, which leads to an increase in their performance as a result of their motivation (52).

The quality of a person's work is strongly related to how satisfied they are with their job. Employees who are satisfied with their occupations are more likely to have a positive outlook on their work, which in turn helps them remain motivated while they are at work.

The ability of an organization to achieve its objectives is strongly linked to the performance of its workforce (53). To some extent, one's professional values, level of commitment to one's work, and ability to contribute to a cohesive workplace environment may all be markers of this characteristic. In this context, both quantity and quality must be taken into account. Additionally, it takes into consideration the output in terms of timeliness and attendance, as well as the efficiency and effectiveness of the job accomplished.

An person or a group's ability to effectively do a task that has been defined and assessed by a supervisor in a firm is referred to as “employee performance”. Standards must be met while using resources in an effective and efficient manner in a changing environment, which calls for adherence to previously specified and accepted standards (54).

## Research methodology

In order to understand the nature of the link between the variables that are the subject of the understudy, a hypothesis study was used in this research. The human resources departments of the various private small family businesses provided the data of their workers, which were then used in this study. The decision to go with private, smaller family businesses was made because these establishments do not provide long-term employment opportunities as public or government-owned companies do. Because of this, they need to place a greater emphasis on the atmosphere of their workplace and the rewards they provide. In this way, businesses will have a better chance of keeping their important staff members in the long term.

The correlational method of inquiry was used for this study since it needed to test hypotheses on the relationships between the different variables. The fieldwork for this study was carried out in its natural setting. Because of this, the research context will be seen as one that was not artificially created. Regarding the completion of the questionnaires, this study has a low level of influence from the researchers toward the participants. Because the data for this research comes from individuals who work for small family businesses, the person serves as the unit of analysis for this particular study. For the purpose of this study, the researcher used a technique known as a cross-sectional study. It entails the investigation of a whole population or a representative sample of the population at a certain instant in time.

In order to obtain data from the staff members working at these small family businesses, the approach of purposive sampling is used. The term “judgmental sampling” may also be used to refer to this procedure. The researcher may be able to obtain data from the responder who is the best fit for the topic at hand, which is one of the reasons why this approach was selected.

The demographic framework for this study is the employee's attendance register, and the way that data is obtained for this research study is *via* the use of the register. In some companies, the researcher was the one who gathered the data. While in other instances the information is gathered with the assistance of pertinent friends in the proper settings.

### Empirical settings and data collection

These investigations were carried out on workers of various private small family businesses in the province of Punjab which's complete data is given in the Table 1. Four hundred questionnaires were sent out to those employees, and roughly 270 questionnaires were returned, resulting in a response rate of fifty-five percent. In order to conduct this research, a trustworthy and comprehensive questionnaire was used. There was a total of 250 replies included for the study, out of which 20 surveys had information that was either missing or incorrect. The demographic profile of the respondents is provided further down.

**Table 1:** Demographic profile of the respondents.

**Demographic profile of the respondents**
**Category**	**Subdivision**	**Frequency**	**Percentage**
Marital status	Married	75	30
	Un-married	175	70
Currently held position	Senior Manager	05	2
	Manager	13	5.2
	Assistant Manager	25	10
	Officer	40	16
	Supervisor	70	28
	Worker	97	38.8
Age	Below 25 years	86	34.4
	25–30	70	28
	31–35	37	14.8
	36–40	30	12
	40 and above	27	10.8
Education	Intermediate	70	28
	Bachelors	130	52
	Masters	40	16
	M.Phil	10	4
	Phd	0	0
Experience	Below 5 years	150	60
	6–10 years	57	22.8
	11–15 years	28	11.2
	16–20 years	10	4
	Above 20 years	5	2
Departments	Marketing	50	20
	Finance	50	20
	Supply chain	50	20
	Human resources	50	20
	Information echnology	50	20

The respondents were employees who worked for a variety of different small companies. The data were acquired both during and after the pandemic scenario *via* the use of a variety of resources in an effort to minimize human interaction to the greatest extent feasible. To approach our target respondents, we used the attendance register maintained by the concerned organization's human resource office, which served as a sampling framework for this study. Every privately held business has various departments, including marketing, sales, finance, supply chain management, human resources, and information technology. As a result, we shall use a methodology known as Stratified Sampling. We may be able to collect data from a representative number of workers in each division if we proceed in this manner.

### Measure and methods

#### Instrument

This research will employ the Mueller-McCloskey Satisfaction Scale (MMSS), created in for social and psychological rewards (1990). This scale has been adjusted and adapted for use in this research of small family businesses' workers since it was originally created to measure nurse employees' satisfaction with their jobs (55). While for measuring the moderating variables which are employee motivation and satisfaction and dependent variable which is an employee performance the scales are adapted and then significantly modified from the research studies of Tremblay et al. (56), Probst (57), and Ramdani et al. (58), respectively. The complete measurement model is run and applied to test the effectiveness of this modified scale and reliability and validity of this instrument is checked as whole in a pooled confirmatory factor analysis test.

#### Confirmatory factor analysis

In order to get reliable and exact answers for all variables, a confirmatory factor analysis must be performed. A pooled CFA analysis will be used in this investigation. To reach the desired level of model fitness, it simultaneously runs all of the latent variables. Since the pooled CFA approach runs all the latent variables concurrently, it is more efficient than Individual CFA (59, 60).

As per the information given in Table 2 the model fit indices show an acceptable fit between the data and the proposed measurement model. The values of the Comparative Fit Index (CFI = 0.938), Root Mean Error of Approximation (RMSEA = 0.049). Chi-square to Degree of Freedom Ratio (x^2^/df = 1.590) are all meeting the cut-off criteria, so the values of the fitness indices meet the excellent standards for model fitness (64–66).

**Table 2:** Pooled CFA model fitness tests.

**Pooled CFA model fitness tests**
**Name of category**	**Name of index**	**Index full name**	**Value in analysis**	**Acceptable value**	**References**
Absolute fit	RMSEA	Root mean square of error approximation	0.070	>0.06	(61)
Incremental fit	CFI	Comparative fit index	0.915	<0.95	(62)
Parsimonious fit	Chisq/df	Chi square/degrees of freedom	2.534	<3	(63)

Pooled CFA Model Fitness TestsAfter running the pooled CFA, it is also necessary to check and verify each item's reliability for further research. CFA of this study's data was used to measure reliability, convergent validity, and discriminant validity. The reliability of the measurement scales was measured with composite reliability, which is preferred to report a scale's reliability (67), a widely used indicator. As per the Table 3 the reliability and convergent validity are accurate and fine because the factor loading of each item is grather than 0.5 which shows the convergent validity of the scale is sufficient and also the combine composite reliability is also above or equal to 0.7 which shows that reliability of the scale is also fine.

**Table 3:** Pooled confirmatory factor analysis (independent, mediating, and dependent variable).

**Pooled confirmatory factor analysis (independent, mediating, and dependent variable)**			
**Scale**	**Items**	**Factor loadings**	**Scale reliability**
Social rewards	The firm provides opportunities for social contact at work.	0.655	0.749
	The firm provides opportunities for social contact with your colleagues after work.	0.831	
	Firm's administration provides opportunities to interact with other disciplines.	0.859	
	Firm's administration provides opportunities to interact with knowledgeable resource.	0.653	
Psychological rewards	You were admired by your immediate supervisor.	0.614	0.700
	You have received recognition for your work from superiors.	0.676	
	You have received recognition for your work from peers.	0.596	
	You have received a fair amount of encouragement and positive feedback on your work.	0.889	
	You have given control over your work setting.	0.729	
Employee motivation	You have given opportunities for career advancement.	0.776	0.784
	You have a given amount of responsibility.	0.778	
	You have given control of your work conditions.	0.805	
Employee satisfaction	I am very satisfied with my organization policies and practices.	0.437	0.708
	I am very satisfied with the social status which my organizations provide to me.	0.761	
	I am very satisfied with the overall compensation which my organizations provide to me.	0.875	
	I am very satisfied with the level of responsibility which my organizations provide to me.	0.759	
Employee performance	My performance is better than that of my colleagues with similar qualifications.	0.732	0.803
	I am satisfied with my performance because it's mostly good.	0.865	
	My performance is better than that of bankers with similar qualifications in other banks.	0.843	
	I would like to perform best for my organization	0.775	

If the measuring scales are different from other measures, they are said to have discriminant validity. HTM analysis was used to test discriminant validity and found that 0.850 was the cut-off for tight discriminant validity and 0.900 the cut-off for liberal discriminant validity (68). Because all of the assessment scales utilized in our research vary from each other, the data used in our study meets discriminant validity standards as per the data given in Table 4 and is eligible for further investigation.

**Table 4:** HTMT analysis.

**HTMT analysis**
	**Social rewards**	**Psychological rewards**	**Employee motivation**	**Employee satisfaction**	**Employee performance**
Social rewards					
Psychological rewards	0.048				
Employee motivation	0.274	0.037			
Employee satisfaction	0.259	0.107	0.406		
Employee performance	0.293	0.051	0.495	0.418	

The shades indicates that the variables are the same and according to HTMT analysis values cannot exist or mention for the same variables.

#### Structural equation modeling

Structural equation modeling (SEM) was used in the Structural model to test the hypotheses, using AMOS 24. As the proposed model contains mediation, the SEM technique was used to analyze all of the paths simultaneously (65, 69, 70). The model fit indices for the structural model are meeting the acceptance criteria as per the information given in Table 5.

**Table 5:** SEM, model fitness tests.

**SEM, model fitness tests**
**Name of category**	**Name of index**	**Index full name**	**Value in analysis**	**Acceptable value**	**References**
Absolute fit	RMSEA	Root mean square of error approximation	0.080	>0.06	(61)
Incremental fit	CFI	Comparative fit index	0.935	<0.95	(62)
parsimonious fit	Chisq/df	Chi square/degrees of freedom	1.534	<3	(63)

### Hypothesis testing

The results of the direct effects in structural model are shown in the Table 6.

**Table 6:** Results of structural model: direct effects.

**Hypothesis**	**Causal path**	***P*-value**	**Standardized estimated**
H^1^	Social rewards → Employee performance	0.685	−0.019
H^5^	Psychological rewards → Employee performance	0.001	0.347
H^9^	Employee motivation → Employee performance	0.001	0.329
H^10^	Employee satisfaction → Employee performance	0.001	0.243

The SEM statistics show that **H**^**1**^ **(Social Rewards → Employee Performance)** is rejected on the grounds of significance level, as the SEM results show that the *P*-values of this hypothesis is not significant. These results suggest that social rewards do not have a direct significant positive impact on employee performance. While **H**^**5**^ **(Psychological Rewards → Employee Performance), H**^**9**^ **(Employee Motivation → Employee Performance), H**^**10**^ **(Employee Satisfaction → Employee Performance)** are accepted on the grounds of significance level, as the SEM results show that the *P*-values of these hypotheses are significant. These results suggest that these variables have a direct significant positive impact on employee performance.

The results of indirect hypothesis which are shown in Table 7 depicts the complete picture of this research study. The study showed that **H**^**2**^ (**Social Rewards → Employee Motivation → Employee performance, β = 0.026**, ***P*** **= 0.082**) is insignificant because the *P*-value is more than 0.05. It also suggests that when organizations provide social rewards to their valuable employees, it does not have any kind of significant impact on the performance of their employees while mediated through employee motivation.

**Table 7:** Results of structural model: indirect effects.

**Results of structural model: indirect effects**					
**Hypothesis**	**Causal path**	**Lower bound**	**Upper bound**	***P*** **-value**	**Standardized estimated**
H^2^	Social rewards → Employee motivation → Employee performance	0.001	0.057	0.082	0.026
H^3^	Social rewards → Employee satisfaction → Employee performance	−0.04	−0.001	0.088	−0.017
H^4^	Social rewards → Employee satisfaction → Employee motivation → Employee performance	−0.018	0.000	0.09	−0.023
H^6^	Psychological rewards → Employee motivation → Employee performance	0.033	0.13	0.005	0.053
H^7^	Psychological rewards → Employee satisfaction → Employee performance	0.03	0.118	0.001	0.044
H^8^	Psychological rewards → Employee satisfaction → Employee motivation → Employee performance	0.014	0.054	0.001	0.063

The study showed that **H**^**3**^ (**Social Rewards → Employee Satisfaction → Employee performance, β = −0.017**, ***P*** **= 0.088**) is also insignificant and suggests that when organizations provide social rewards to their valuable employees, it does not have any kind of significant impact on the performance of their employees while mediated through employee satisfaction.

For another hypothesis which is **H**^**4**^ (**Social Rewards → Employee Satisfaction → Employee Motivation → Employee performance, β = −0.023**, ***P*** **= 0.09**) is also insignificant and suggests that when organizations provide social rewards to their valuable employees, it does not have any kind of significant impact on the performance of their employees while mediated through employee satisfaction and then employee motivation simultaneously.

The study showed that **H**^**6**^ (**Psychological Rewards → Employee Motivation → Employee performance, β = 0.053**, ***P*** **= 0.005**). This hypothesis is also significant because the *P*-value is <0.05. It suggests that when organizations provide psychological rewards to their valuable employees, it does have significantly positive impact on the performance of its employees while mediated through employee motivation.

The second hypothesis which is relevant to the psychological rewards is **H**^**7**^ (**Psychological Rewards → Employee Satisfaction → Employee performance, β = 0.044**, ***P*** **= 0.001**). This hypothesis is also significant because the *P*-value is <0.05. It suggests that when organizations provide psychological rewards to their valuable employees, it does have significantly positive impact on the performance of its employees while mediated through employee satisfaction.

For last hypothesis which is relevant to the psychological rewards is **H**^**8**^ which is determining the results of (**Psychological Rewards → Employee Satisfaction → Employee Motivation → Employee performance, β = 0.001**, ***P*** **= 0.063**). This hypothesis is also significant because the *P*-value is <0.05. It suggests that when organizations provide psychological rewards to their valuable employees, it does have significantly positive impact on the performance of its employees while mediated through employee satisfaction and then employee motivation.

## Discussion

Small family businesses may secure the performance of their employees using psychological incentives while considering employee motivation and pleasure as a mediating component. Considering the findings of this research, we might say that organizations are still reeling from the financial toll that COVID-19 has taken. COVID-19 findings are supported by earlier investigations, despite the lack of COVID-19 circumstances (71, 72). Another study done by Ma et al. (73) suggests that psychological rewards played a vital role in changing employee behavior toward their organization. They remain loyal to their organizations and their performance continuously upsurges. It is also suggest by De Gieter et al. (74) and Hinds (75) that employee performance resulted through the enhanced employee satisfaction and motivation lead toward the low turnover ratio ensures they have a high level of employee performance, leading to customer satisfaction.

While looking into the results, the answers related to the social rewards are interesting as well as worth noting. The study suggests that the social rewards have insignificant impact on the employee performance while mediated through employee motivation and employee satisfaction. These kinds of results require more in-depth approach to understand the scenario in which the results are gathered. As discussed by Anderson (76), Berman et al. (77), Heerey (78), and Rademacher et al. (79) the social rewards could have a different impact on different respondents in a different geographical, economic and cultural scenario. Social rewards are considered as non-monetary rewards which could be considered as viable solution to boost the performance of employees in an organization while considering this fact that they are already receiving appropriate monetary benefits (80, 81). Introducing social rewards to enhance the employee performance through motivation and satisfaction, in presence of inappropriate monetary benefits could be considered as a non-functional approach in some cases.

## Conclusion

It is concluded from the above discussion and findings that small family firms which could be considered as a basic and essential part of any countries' economy is primarily based upon the performance of its employees. If employees are unable to perform well then the organization will not be able to sustain its self in a longer run. It is imperative to keep employees happy and loyal with their organization to work more in the same organization (82), thus generating more productivity and successful work. To complete that kind of task, in a post COVID-19 scenario when organizations are still fighting to maintain sustainable financial stability to run the firm smoothly. They are somehow forced to evaluate the non-monetary rewards in connection with the employee performance due to their lack of financial stability. While the most discussed non-monetary rewards are the social and psychological rewards. According to this study findings, the psychological rewards will help the organizations keep their employee satisfied and motivated enough so they could perform really well for the organization (9, 11). While applying social rewards for the respondents with these psychographic and geographic credentials are not helpful at all. Investing any kind of resources which could include time or research to enhance employee performance will go in vain. It could be possible to apply social rewards in any other psychographic and geographic scenario with positive and significant results, which could lead toward a more accurate path that how the organizations could keep the performance of their employees elevated while considering different non-monetary rewards on their disposal (83).

## Managerial implications of the study

This research might also be used to examine the behavior of huge corporations and organizations, such as schools, banks, and airlines. Managers will benefit from this information as they reexamine their management policies related to their employee's management. In order to improve productivity and increase employee loyalty, they may implement a more comprehensive and realistic non-monetary incentives system in their company. Manager, could learn more about the psychological incentives which are needed by the employees of their organizations through this research study. This study also provides guidance to the management of different organizations which have the employees belongs to the demographic profile of the respondents used for this study. In this way they could be able to apply more focus on providing the psychological incentives to its employees rather than focusing upon the social rewards, which according to this study don't have any positive impact on the employee performance through satisfaction or motivation.

## Limitations of the study

Because this study was undertaken particularly for small family businesses in a single province, it is unable to be applied to other regions. The study's geographic scope might potentially be seen as a drawback. This research is also constrained by a lack of funding and time. It's also feasible that the findings might vary dramatically over a short period of time of COVID-19 pandemics. Because of their busy schedules, it's possible that workers won't be able to concentrate on this questionnaire. There's no way around it: this restriction is correct. Because of the extreme level of stress they are under in the COVID-19 scenario.

Additionally, they must take care of their personal and family affairs during these frantic times. As a result, gathering the study's data was a very difficult task. In this survey, participants fill out a questionnaire that has been validated, authenticated, and shown trustworthy. The majority of respondents said it was well-written and easy to grasp.

## Data availability statement

The datasets presented in this article are not readily available because of the privacy of its respondents. Requests to access the datasets should be directed to MS, waqas_sadiq2011@hotmail.com.

## Author contributions

MS, JH, and CH: conceptualization. MS and MA: data curation. MS and JH: formal analysis, validation, and writing—review and editing. CH and MA: methodology and supervision. MS, JH, and MA: writing—original draft. All authors have read and approved the final version of the manuscript.

## Funding

This study was supported by the National Social Science Foundation of China (No. 21BGL047).

## Conflict of interest

The authors declare that the research was conducted in the absence of any commercial or financial relationships that could be construed as a potential conflict of interest.

## Publisher's note

All claims expressed in this article are solely those of the authors and do not necessarily represent those of their affiliated organizations, or those of the publisher, the editors and the reviewers. Any product that may be evaluated in this article, or claim that may be made by its manufacturer, is not guaranteed or endorsed by the publisher.
